# Clinical performance of a shielded diode for small field relative dosimetry in a 1.5 T MRI‐linac using measurements and simulations

**DOI:** 10.1002/mp.70356

**Published:** 2026-02-19

**Authors:** William S. Ferris, Damian Czarnecki, Joel J. St‐Aubin, Daniel E. Hyer

**Affiliations:** ^1^ Department of Radiation Oncology University of Iowa Iowa City Iowa USA; ^2^ University of Applied Sciences, Technische Hochschule Mittelhessen Giessen Germany

**Keywords:** 1.5 T, MR‐linac, small field

## Abstract

**Background:**

Small field dosimetry on magnetic‐resonance (MR) guided linear accelerators (linacs) has become increasingly more important for accurate beam modeling due to the increase in the number of patients and variety of disease sites treated on MR‐linacs such as those with small fields. Dosimetry in magnetic fields has been proven to be difficult due to the Lorentz force and intrinsic detector responses. For example, there is evidence in the literature that shielded diodes may cause a shift in the shape of the lateral dose distribution. However, there is no work in the literature investigating the shape change for the Sun Nuclear EDGE shielded diode.

**Purpose:**

The goal of this research was to use measurements and simulations to compare the performance of the EDGE diode to two other scanning detectors and film for small field relative dosimetry in a linac with an external magnetic field.

**Methods:**

The detectors investigated in this work were the EDGE detector, the PTW 60019 microDiamond, the PTW 31021 Semiflex 3D, and Gafchromic EBT3 radiochromic film. The PTW Trufix system was used to align the sensitive volume of each detector to the radiation isocenter. To make this possible, a custom holder was manufactured for the EDGE detector which was compatible with Trufix. To enable comparison, the detector profile measurements were reproduced by Monte Carlo (MC) simulations. To simulate radiation transport in the measurement setup, detailed MC models of the examined detectors were built, together with a model of a 1.5 Tesla (T) Elekta Unity linac.

**Results:**

Radiochromic film was used as the ground truth for profile shape in this work. The MC beam spot size and collimation offset were tuned to match the film results. Profile shape was consistent between measurements and MC simulations for each of the scanning detectors. The EDGE detector was found to under‐represent the effect of the magnetic field on profile shape, which is consistent with findings of other shielded diodes in the literature. Repeated profiles indicate that the intra‐type variation of the active volume positioning is about 0.6 mm. The Semiflex 3D ionization chamber over‐represents the effect of the magnetic field on profiles. The microDiamond provided the best agreement to film and MC for small fields based on the profile, percent depth dose (PDD), and output factor (OF) results.

**Conclusions:**

This study reinforces the importance of detector choice for characterizing small fields in the presence of magnetic fields. The EDGE detector was shown to misrepresent the shape of the crossline profile for small fields in a 1.5T magnetic field. Film is recommended for determining the dose distribution shape, but it is difficult to position precisely and thus cannot be used to determine the lateral shift with high precision. The microDiamond detector was determined to most accurately characterize both the lateral shift and shape of the lateral dose distribution of the detectors investigated. The investigated ion chamber and shielded silicon diode should be used with caution for small fields in the presence of magnetic fields due to perturbances to profile shape.

## 1 INTRODUCTION

The Unity (Elekta AB, Stockholm, Sweden) is a 7 megavolt (MV) flattening filter free (FFF), 1.5 tesla (T) magnetic resonance (MR) guided linear accelerator (linac) that specializes in adaptive radiotherapy.^1^ The magnetic field is in the longitudinal direction (perpendicular to the beamline), which creates a Lorentz force on the charged particles in the transverse plane. Quantifying the dose distributions in this magnetic field, especially for small fields, has proved challenging due to the electron return effect and the lateral shift of the dose distribution due to the Lorentz force.^2^, ^3^, ^4^, ^5^

As the number of disease types treated on MR‐linacs increases, there is more interest in examining detectors for small field relative dosimetry in the presence of MR fields. For example, the department of radiation oncology at the University of Iowa has recently started treating stereotactic body radiation therapy (SBRT) on the MR‐linac, which typically uses fields as small as ∼1.4 × 1.4 cm^2^ (i.e., two MLC leaf widths). The smallest field possible on the Unity is ∼0.5 × 0.5 cm^2^ but is not typically used clinically at our institution. Proper commissioning of the profile shape and lateral position relative to the mechanical isocenter is important for these small fields. Accurate characterization requires both measurements and Monte Carlo (MC) simulations. For example, measurements are susceptible to detector manufacturing variations and setup error, and simulations are dependent on accurate material/geometry and beam model definitions.

At the University of Iowa, we use the EDGE detector (Sun Nuclear Corp., Melbourne, FL) to characterize dose distributions of small fields for standard linacs without magnetic fields. However, the response of the EDGE in MR fields was unquantified at the time of commissioning the MR‐linac, therefore commissioning measurements for small fields on the MR‐linac were instead performed using a PTW microDiamond (PTW Dosimetry, Freiburg, Germany).^2^ There are instances in the literature of the EDGE detector being used in MR fields,^6^, ^7^, ^8^ but only in lower field strengths such as 0.35 T and not for relative scanning profile measurements in small fields.

O'Brien et al. performed relative dosimetry on a Unity linac with several ion chambers, diodes, and a microDiamond and showed that shielded diodes may misrepresent the shape of the profile in the direction of the magnetic field shift.^2^ However, the diodes included in that study were the PTW 60018 and PTW 60016, and not the EDGE detector. The EDGE is constructed from different materials and has a “pancake” geometry instead of a cylindrical geometry like the PTW diodes. Therefore, the response to a magnetic field may be different. In addition, O'Brien et al. performed only measurements rather than simulations, and each detector was centered in the bore separately with MV images. Yano et al. simulated small field dose distributions with MC to compare to the measurement results of O'Brien et al., but the EDGE detector and the PTW microDiamond were not included.^5^

The purpose of this work was to use measurements and simulations to compare the performance of the EDGE detector to other detectors commonly used for small field relative dosimetry on the Unity MR‐linac. An emphasis is placed on quantifying the lateral shift and shape change of the profiles for each detector, which requires simulations and an absolute positioning system with a known geometric relationship.

## 2 METHODS

Four detectors were investigated which are common detectors used for small field dosimetry in nonmagnetic fields: an EDGE detector, a PTW microDiamond detector (PTW‐60019), a PTW Semiflex 3D ion chamber detector (PTW‐31021), and EBT‐3 film (Ashland Advanced Materials, Bridgewater, NJ). These detectors will henceforth be referred to as EDGE, microDiamond, Semiflex 3D, and film, respectively. Scintillation detectors, despite their high spatial resolution and low energy dependence, were not considered for this work because they are not compatible with the PTW Beamscan MR tank and they have been shown to have complex responses to applied magnetic fields.^9^ Film was assumed to be the closest detector to ground truth in terms of profile shape due to its high resolution, water‐equivalency, and minimal perturbance in MR fields.^3^

MC simulations were conducted to complement and support the experimental findings. Given that the experimentally measured detector response was directly compared with the results from simulations, detailed profile fitting of the virtual radiation source could be considered unnecessary. However, to minimize any impact on the detector response that could result from a different dose profile, we matched the measured and MC simulated profiles. The MC model is a measurement‐driven model rather than a direct simulation since the exact materials and components of the linac are not precisely known.^10^ Therefore, measurements with film were used to optimize the MC model to match the shape of the dose profiles. The scanning data measured in a water tank included percent depth doses (PDDs), uncorrected output factors (OFs), and crossline/inline profiles.

### 2.1 Water tank measurements

**FIGURE 1:**
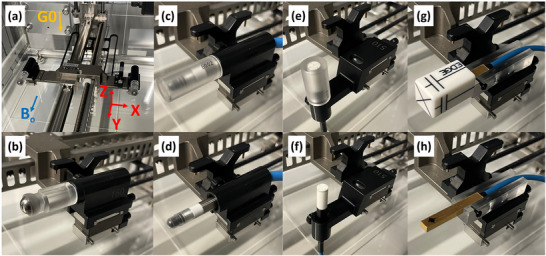
The PTW water tank (a) and close‐up of the alignment BB (b). The Semiflex 3D (c, d), microDiamond (e, f), and EDGE (g, h) with the alignment cap (c, e, g) and after removing the cap prior to measurement (d, f, h). The alignment jigs place the EPOM of each detector at the center of the BB.

A PTW Beamscan MR water tank and the PTW Trufix positioning system were used for all scanning measurements and OFs. The source to axis distance (SAD) of the Unity is 143.5 cm. Profiles were acquired at gantry zero, 133.5 cm source‐to‐surface distance (SSD), and depths of 5 and 10 cm. The field sizes measured were 1, 2, 3, 5, and 10 cm^2^. OFs were acquired at 133.5 cm SSD, 10 cm depth, 100 monitor units (MU), with an average of three measurements. OFs were measured along the central axis (geometric center of the square field) and corrected to the peak profile position using the crossline profile. Measurements were only performed for one detector orientation with respect to the magnetic field, with the orientations shown in Figure 1. The orientation with the least perturbations to the dose distribution, or the recommended orientation, was chosen for each detector.^11^ The measured profiles (PDD, crossline, and inline) were not post‐processed other than normalization to preserve profile shape. Dwell times were set such that noise in the profiles was small and smoothing was not necessary.

Absolute positioning within the radiation field was performed using the Trufix system. With the Trufix system, a metal ball‐bearing (BB) was first aligned to the radiation isocenter with orthogonal planar MV images (0.4 mm pixels, 0.217 mm at iso). The BB has a precisely known position relative to the scanning arms. Then, the BB was removed and positioning jigs specific to each detector model were used to place the effective point of measurement (EPOM) of the detector at the same location as the center of the BB. At the time of this work, there was no commercially available positioning jig specific to the EDGE detector; therefore, one was designed and manufactured in‐house for this work. The dimensions of the EDGE were obtained from the manufacturer. The custom EDGE holder is shown in Figure 1 (g) and (h). Alignment caps are used to aid in positioning the detectors within the jig but are removed prior to measurement. The uncertainty in the dimensions of the Edge Trufix holder from the milling machine is estimated to be 0.002–0.004″ (∼0.05–0.10 mm) and the uncertainty of the Trufix positioning system is 0.1 mm.^12^

To investigate the reproducibility of the active volume between detectors of the same model, profiles were measured with three EDGE detectors (SN‐6429411‐2010‐05, SN‐69457006‐2011‐02, SN‐W205526012) using the same positioning and field size (1 × 1 cm^2^, as produced with jaws and MLC). The absolute position of the outer housing of the detectors was the same, therefore any differences in profile position can be attributed to the position of the active volume within the housing.

Uncorrected OFs (ratios of charge collected at various field sizes) were measured using a detector positioned along the central axis (CAX) of the beam (geometric center of the square field), denoted OF_center_. Three measurements were performed with 100 MU each and the setup conditions were 133.5 cm SSD and 10 cm depth. However, the magnetic field causes a shift in the peak position of the crossline profile, especially for small fields. Therefore, the ratio of the peak value to the value along the CAX was obtained for each crossline profile, and OF_peak_ was calculated by applying this ratio to the charge collected. Uncorrected OFs (ratios of charge collected) are presented rather than fully corrected OFs (ratios of doses) to highlight the differences in response among the different detectors.

### 2.2 Film dosimetry

Radiochromic film has the advantages of minimal energy dependence for MV beams, high spatial resolution, and minimal perturbance on the beam.^13^ EBT‐3 film has been shown to be a suitable detector for relative dosimetry in the presence of a 1.5 T magnetic field.^3^, ^14^, ^15^ Film was assumed in this study to be the closest to the “ground truth” dose distribution in terms of profile shape. However, absolute positioning of film with submillimeter accuracy was not possible. Therefore, film was not used for analysis of the lateral shift. Profiles were measured using film to determine the shape of the dose distribution in the inline and crossline directions. The film was placed in solid water HE (Sun Nuclear Corp.) on the treatment table at a height to mimic the position of the profiles measured in the water tank and the same water‐equivalent depth as the water tank measurements. Solid Water HE was assumed water equivalent based on manufacturer‐specified electron density ratio to water of 1.000.

An optical density (OD) to dose calibration curve was created using a Versa HD linac (Elekta AB, Stockholm, Sweden) using a 6 MV energy. A conventional linac was used for calibration because radiochromic film has been shown to have a minimal energy dependence^13^ and a flat field and accurate geometric setup were easier to achieve. EBT‐3 film from batch 9072202 was used for all film testing. Nine calibration films were irradiated in solid water to nine known dose levels ranging from 0 to 400 cGy. The measurement films on the Unity were irradiated to receive approximately 300 cGy in the center of the field. Since the magnetic field causes a shift in the dose distribution in the crossline direction, the sagittal laser was marked on the film to provide the location of the inline profile. The calibration curve and all film analysis in this work were performed using the Film QA Pro software (Ashland Advanced Materials). Triple channel dosimetry was performed to remove non‐uniformities in the thickness of the active layer of the film and other non–dosimetric responses of the film.^13^ All films were scanned 24.0 ± 0.5 hours after irradiation. The scanner was warmed up prior to scanning, and the scanning orientation was kept constant. The scan resolution was set to 100 dpi, or 0.254 mm spacing, and profiles were averaged over a ∼5 mm wide region (20 pixels).

**FIGURE 2:**
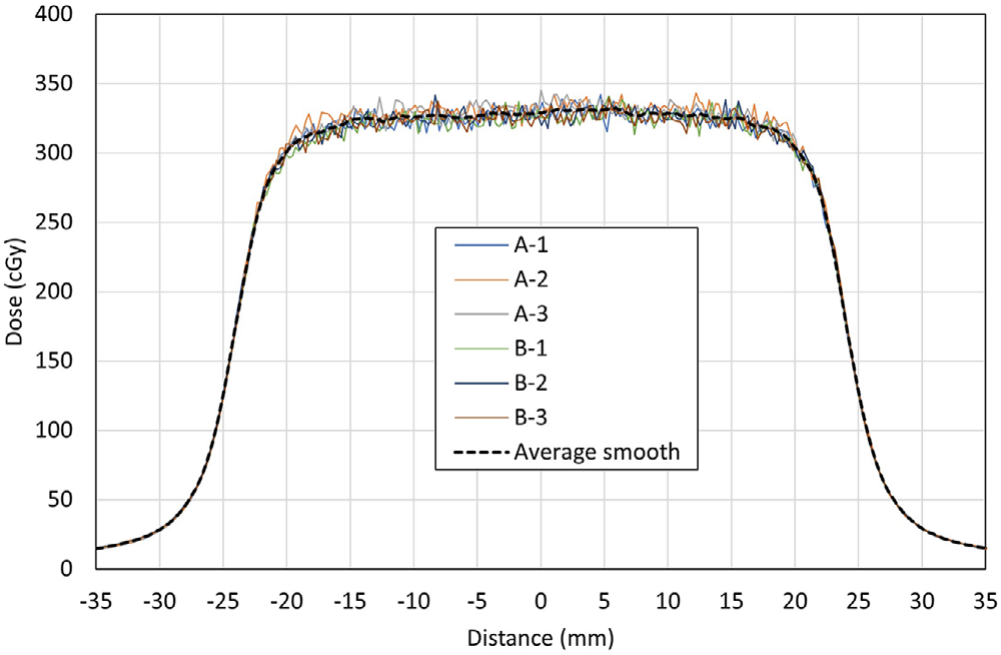
Example of the raw film profiles registered at the 50% penumbra and the final smoothed and averaged profile for 5 × 5 cm^2^, 5 cm depth, and inline. A and B represent different irradiated films, and the numbers represent three repeated scans on different locations of the scanner bed.

Additional steps were taken to ensure accurate film dosimetry. For each profile (field size and depth), two films were irradiated to provide an average. In addition, each film was scanned three times on different locations of the scanner bed. The six resulting profiles were then spatially registered at the 50% penumbra, separately smoothed using a moving average filter of 5 data points (∼1 mm) and averaged together. Figure 2 shows an example of the raw films and the final averaged profile.

### 2.3 MC simulations

All MC simulations in this work were carried out using the EGSnrc coding system^16^ The main MC simulation settings are summarized in Table 1, adhering to the guidelines suggested by Task Group 268 of the American Association of Physicists in Medicine (AAPM).^17^ The enhanced electric and magnetic field (EEMF) macro, developed by Malkov and Rogers^18^ was utilized in this work to model particles traveling in an external magnetic field.

**TABLE 1:** An overview of the main characteristics of the MC simulations used in this study.

Item name	Description
Code, version	EGSnrc code system, release v2023^11^
Validation	Measurements against simulations
Timing	2E8 histories, 200 CPUs, 5900 h (CPU time) per dose profile simulation.
Transport parameters	Photon cutoff energy = 10 keV. Electron cutoff energy = 521 keV
Geometry	Linac head model, detectors placed in a 30 × 30 × 30 cm^3^ water phantom
Scoring	Dose‐to‐water, dose‐to‐detector in‐water
Statistical uncertainties	Central field < 0.15 %, penumbra < 0.3 %, out‐of‐field < 2%
Statistical method	History‐by‐history

#### 2.3.1 Radiation source

The BEAMnrc user code of the EGSnrc code system was used to simulate the radiation field of an Elekta Unity MR‐linac.^19^ To create a realistic radiation field of a 7 MV FFF Elekta Unity, we adapted the approach from Yano et al.^5^ The 6 MV Elekta Precise linac head model, which has been examined and verified in earlier research,^20^ was used to develop an MR‐linac head model. Following the work by Yano et al.,^20^ the energy of the electrons hitting the Bremsstrahlung target was set to 7 MeV and the flattening filter of the linac was replaced by a brass plate to adjust the photon energy. In this approach, the brass plate thickness can likewise be used to modify the cryostat's impact on the radiation field. As shown by Yano, a thickness of 3.4 cm of brass in the radiation field provided the best agreement with the radiation field of a 7 MV Unity linac. Moreover, the incident electron beam size of the linac head model was modeled as a Gaussian intensity distribution and the full‐width half maximum (FWHM) of the Gaussian was optimized to match the shape of the film measurements. Directed bremsstrahlung splitting (DBS), a variance reduction technique, was applied to increase the MC simulations' efficiency. For a 3 × 3 cm^2^ radiation field, the DBS technique was employed with a splitting number of 1500 and a splitting field radius of 4 cm at 133.5 cm from the source.

#### 2.3.2 MC simulations in water phantom

**FIGURE 3:**
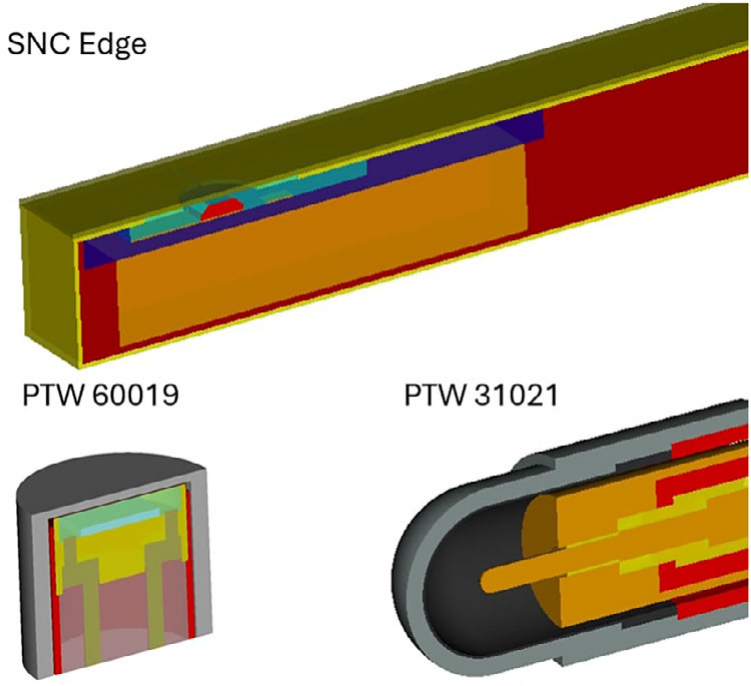
Detector cross sections used in this study; not to scale. Various materials are represented in distinct colors.

The EDGE detector and the microDiamond detector were modeled using the geometry modules of the EGSnrc C++ class library, according to the technical drawings provided by the manufacturer. The MC‐based model of the Semiflex 3D ionization chamber used in this work was investigated in prior studies.^21^, ^22^ Cross sections of the three detector models are shown in Figure 3.

All detectors were placed in a 30 × 30 × 30 cm^3^ cubic water phantom. The dose in the sensitive volume of the ionization chambers and the absorbed dose to water in a small cuboid water voxel (1 × 1 × 2 mm^3^) were determined using the EGSnrc application egs_chamber.^23^ To improve lateral resolution, the voxel dimensions in the lateral directions were set to 1 mm. At a depth of 5 cm—where the dose profile has no significant curvature—the voxel height was set to 2 mm to increase the efficiency of the simulation. The variance reduction methods provided in egs_chamber were utilized to efficiently calculate dose. The photon cross‐sectional enhancement technique (XCSE) with an enhancement factor of 128 was used in the detector geometry with a slightly larger region surrounding the detector. To reduce the simulation time of electrons, the Russian Roulette variance reduction technique with a survival probability of 1/256 was used. Furthermore, all detector positions required for a dose profile calculation were computed in a single simulation run with the use of intermediate phase space scoring.

The MC simulations were intended to support the findings of the measurements, not to provide perfect agreement with the measurements such as when commissioning a beam model. For example, simulations can indicate profile shape differences between detectors while removing measurement uncertainties. Therefore, the simulations were used in a qualitative and visual manner, rather than quantitative.

## 3 Results

Measurements were acquired for field sizes ranging from 1 × 1 cm^2^ to 10 × 10 cm^2^. The detector response variations due to the external magnetic field appear in regions with a high dose gradient, such as field edges, and this behavior is consistent across all field sizes. The 3×3 cm^2^ field provides a clear, well‑defined penumbra to illustrate these effects. Larger field sizes mainly extend the central dose plateau, where all detectors agree well, while smaller field sizes do not have a well‐defined penumbra and plateau region due to the source occlusion effect. Therefore, 3 × 3 cm^2^ was chosen as a representative field size for presentation of the results.

**FIGURE 4:**
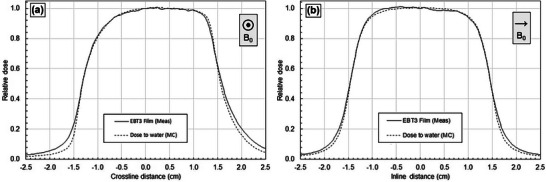
Comparison of EBT3 film and dose‐to‐water calculated with MC for 3 × 3 cm^2^ crossline (A) and inline (B) profiles at 133.5 cm SSD and 5 cm depth. The film was used to tune the MC model, and the final MC model is shown. The direction of the magnetic field is indicated.

Figure 4 compares the measured EBT3 film to dose‐to‐water calculated with MC. The MC model was tuned to match the measured film profiles. Specifically, penumbra shape was adjusted via the width of the target spot size FWHM, and the field size was adjusted via the collimation offset (an effective gap between opposing collimators to account for transmission through the leaf curvature). Figure 4 results indicate profile shape agreement between film and the final MC model. There are minor disagreements in the tail region as tuning was focused on the penumbra shape and field size. The film was not absolutely positioned in the bore and was thus only used for determining the *shape* and not the lateral position. In addition, the MC has less noise than film. Therefore, MC is more useful for isolating effects from detector materials and geometry without setup errors and noise.

**FIGURE 5:**
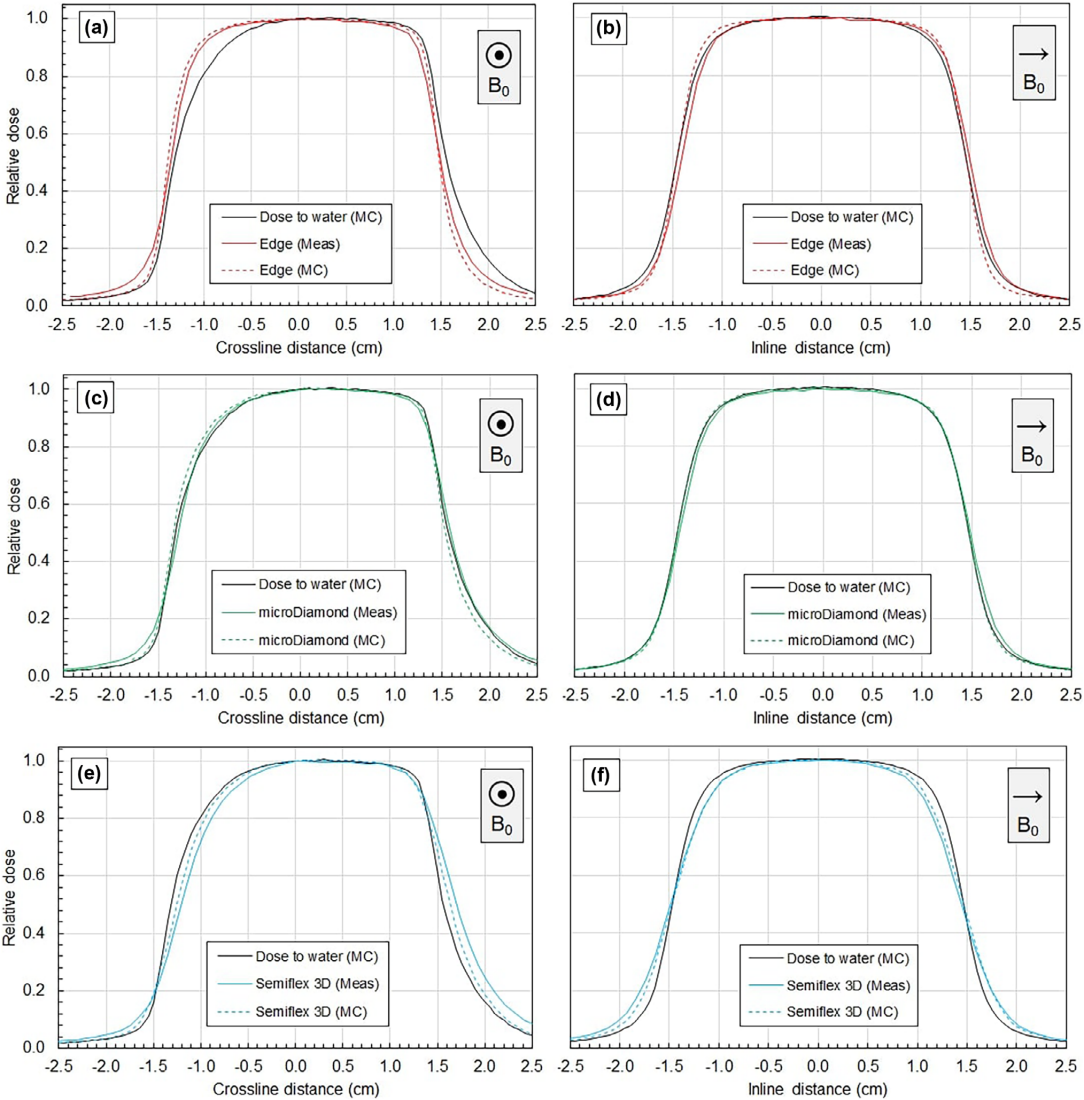
Crossline and inline profiles for a 3 × 3 cm^2^ field at 133.5 cm SSD and 5 cm depth. Scanning detector measurements are presented without shifts, using absolute positioning of the Trufix system.

Figure 5 shows crossline and inline profiles for a representative small field at 5 cm depth for each scanning detector. Both the measured profile and the MC‐simulated profiles are shown, which indicate good agreement between the measured and MC results for each detector. The EDGE detector and the Semiflex 3D were observed to under‐ and over‐represent the lateral shift of the profile in the crossline direction (Figure 5), respectively. These trends are present for all field sizes, but the magnitude of the effect is amplified at small fields such as 3 × 3 cm^2^. For the EDGE, the effect is only observed in the crossline direction (as a profile shoulder shape change), or the direction of the magnetic field shift. The EDGE accurately characterizes the profile shape in the inline direction, suggesting that the effect in crossline is due to the interference of the magnetic field, and not an intrinsic detector effect such as volume averaging. Conversely, the ion chamber suffers from volume averaging, a crossline profile lateral shift, and a crossline profile shape change. The microDiamond detector provides the best agreement with the MC dose‐to‐water based on profile width and shape.

The CAX shifts of the crossline and inline profiles are shown in Table 2, which were calculated as the center of the profiles at 50% relative dose. The CAX shift was found to have little dependence (< 0.25 mm) on field size and depth for a given detector for field sizes between 1 × 1 cm^2^ and 10 × 10 cm^2^. Therefore, the data in the table are presented for 3 × 3 cm^2^ as a representative small field. The CAX shifts as determined by MC dose‐to‐water were 1.23 mm in crossline and 0.00 mm in inline. The microDiamond detector was the closest detector to accurately represent this CAX shift, with an average crossline shift of 1.41 mm (range 1.32–1.53 mm) across field sizes 1 × 1 cm^2^ and 10 × 10 cm^2^. The Semiflex 3D consistently over‐represented the true lateral shift of the crossline profile at all field sizes, with an average crossline CAX shift of 2.46 (2.33–2.56 mm). The EDGE consistently under‐represented the lateral shift of the crossline profile at all field sizes, with an average crossline CAX shift of 0.67 (range 0.60–0.77 mm). All measured inline CAX shifts were less than ± 0.5 mm from center.

**TABLE 2:** CAX shifts calculated at the 50% penumbra of 3 × 3 cm^2^ profiles at 5 cm depth. The MC data is bolded to indicate it is the reference ground truth value, and relative delta values are displayed. Scanned measurement data were positioned with the Trufix positioning system and have not been post‐processed other than normalization.

	Crossline (mm)	Crossline Δ (mm)	Inline (mm)	Inline Δ (mm)
Dose‐to‐water (MC)	1.23	0.00	0.00	0.00
microDiamond (MC)	0.96	−0.27	0.00	0.00
microDiamond (Meas)	1.46	0.23	0.21	0.21
EDGE detector (MC)	0.51	−0.72	0.00	0.00
EDGE detector (Meas)	0.73	−0.50	0.41	0.41
Semiflex 3D (MC)	1.91	0.68	−0.02	−0.02
Semiflex 3D (Meas)	2.48	1.25	−0.23	−0.23

The expanded uncertainty (*k* = 1) of the absolute positioning of the detectors within the field is estimated to be 0.259 mm, which is derived from stepper accuracy (0.1 mm), alignment jig accuracy (0.1 mm), and the resolution of the MV images for positioning (0.4 mm at panel, 0.217 mm at iso). The positioning of the active volume within the detector housing (e.g., manufacturing uncertainty) also contributes to uncertainty, but is unknown and different for each detector type. Due to these uncertainties, the MC data is more valuable for determining relative shift magnitudes. Both the measured and MC data indicate that the microDiamond provides the most accurate crossline CAX shift with respect to the MC dose‐to‐water. In the inline direction, the measured CAX shift was less than 0.1 mm for all MC profiles, and less than 0.45 mm for all measured profiles for all field sizes (1 × 1 cm^2^ to 10 × 10 cm^2^), depths (5 and 10 cm), and detectors.

**FIGURE 6:**
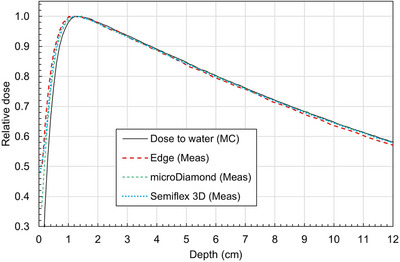
PDDs measured with each scanning detector for a 3 × 3 cm^2^ field at 133.5 cm SSD. Values are displayed after accounting for EPOM shifts.

Figure 6 shows the measured PDDs for each detector for a representative small field. The PDDs at 10 cm depth for this field were 62.9%, 64.5%, 64.1%, and 64.4% for the EDGE, Diamond, Semiflex 3D, and MC dose‐to‐water, respectively. The depth of the EDGE detector was set based on the manufacturer's stated distance below the face of the detector. The PDD from the EDGE detector appears to be slightly shallower than that of the microDiamond or Semiflex 3D. However, the PDDs among the three detectors are very similar in shape.

**FIGURE 7:**
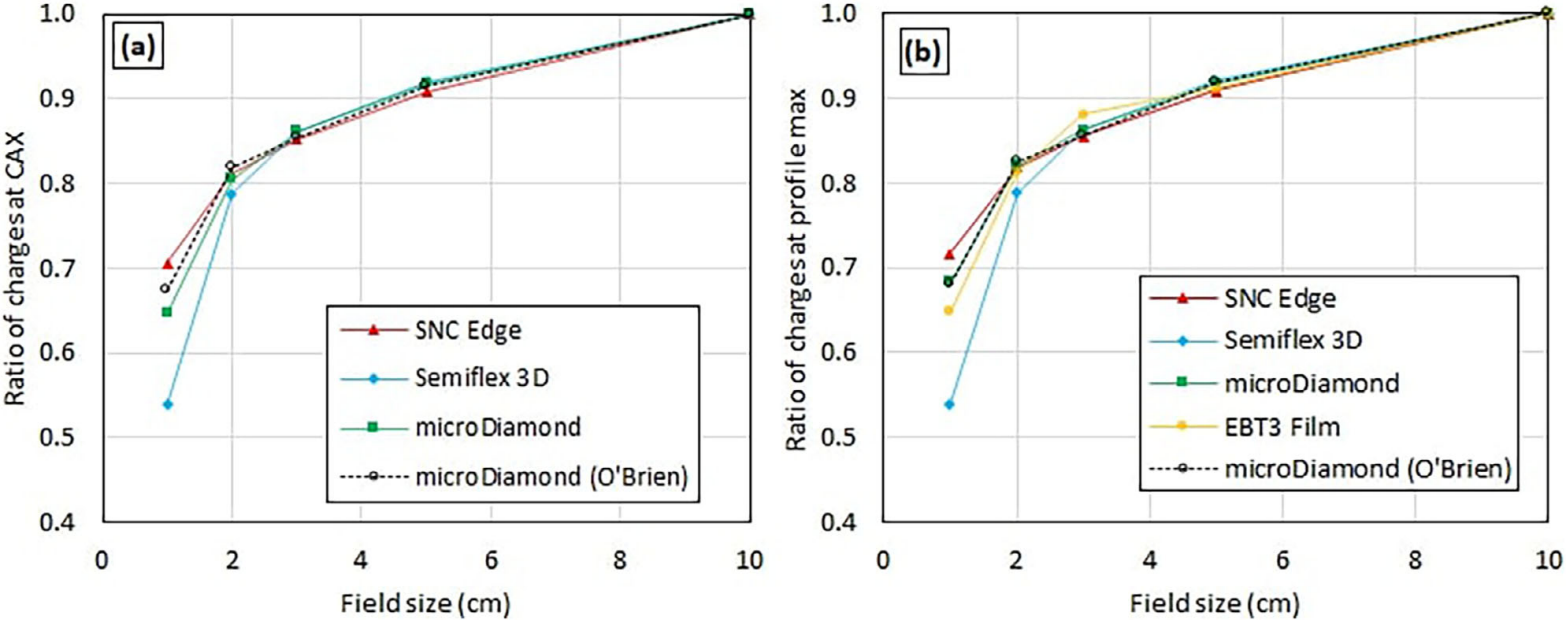
OFs (ratio of charges) compared to a 10 × 10 cm^2^ field collected (a) along the CAX (OF_center_) and (b) at the profile maximum (OF_peak_) at 10 cm depth and 133.5 cm SSD. Only the OF_peak_ was measured with film since the film was not absolutely positioned in the bore. Values from O'Brien et al. reproduced with permission.^2^ ^.^

Figure 7 shows the OFs measured between small fields and the 10 × 10 cm^2^ field. Note: the term “output factor” in this work is defined as the ratio of charges; see the discussion section. For field sizes 2 × 2 cm^2^ and larger, the OFs differ by 3.5% or less among the detectors in this work. However, at the 1 × 1 cm^2^ field size, the Semiflex 3D chamber underestimates OF_center_ by 10.7% and OF_peak_ by 14.4% relative to the microDiamond. The EDGE detector overestimates OF_center_ by 6.0% and OF_peak_ by 3.4% at that field size relative to the microDiamond. The film and microDiamond OF_peak_ results agree within 3.5% for 1 × 1 cm^2^. The microDiamond values agree with those measured by O'Brien et al. within 2.8% for OF_center_ and 0.4% for OF_peak_.^2^ The microDiamond is considered the reference measurement due to the consistent results with the literature and the smallest reported small field correction factors.^24^

**FIGURE 8:**
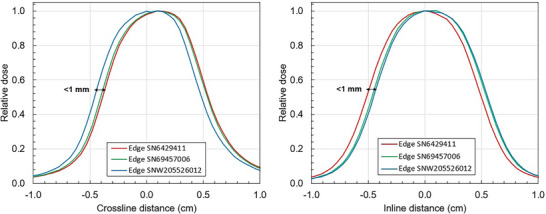
Crossline and inline profiles for a 1 × 1 cm^2^ field at 133.5 cm SSD and 5 cm depth measured with three different EDGE detectors to investigate variation in active volume placement.

Profiles were measured with three EDGE detectors of the same model number but different serial numbers to investigate variation in active volume positioning between detectors, shown in Figure 8. The analysis was performed for a 1 × 1 cm^2^ field to maximize sensitivity of lateral shifts. The measurements were performed sequentially using the same detector holder and coordinate system. Therefore, any crossline and inline CAX centering variation can be attributed to active volume positioning within the housing. The shape of the profile was consistent between the different SNs. Among the three detectors, the CAX shift varied by 0.59 to 0.66 mm in both the crossline and inline directions and two depths, indicating a variation in active volume positioning of approximately 0.6 mm in each direction.

## 4 Discussion

The results indicate that the microDiamond detector provides the best agreement to MC dose‐to‐water of the three scanning detectors investigated in this work. The profile shape and CAX shifts were the closest to the MC simulations and film. The EDGE detector under‐represents the shift due to the magnetic field in the crossline direction, which was confirmed by simulations. The Semiflex 3D ion chamber suffers from volume averaging effects and a lateral shift that are apparent at small field sizes (< 5 × 5 cm^2^). The CAX shift magnitudes observed for Semiflex 3D and EDGE detectors are much larger than the positioning uncertainty of the Trufix system. These conclusions agree with findings in the literature on ion chambers and diodes in magnetic fields. The PDDs were found to be less affected by choice of detector. However, the PDD results from the EDGE detector were the outlier compared to the microDiamond and Semiflex 3D detectors.

The data indicate that the OF should be measured at the peak position of each profile instead of along the CAX since the OF_center_ is sensitive to changes in the dose distribution. For example, the difference between the EDGE and the microDiamond for a 1 × 1 cm^2^ field was 6.0% for OF_center_ and 3.4% for OF_peak_. In addition, the difference between the value in this work and the work of O'Brien et al.^2^ for the microDiamond for 1 × 1 cm^2^ was 2.8% for OF_center_ and −0.4% for OF_peak_. OF_peak_ is less sensitive to setup differences as minor shifts of the CAX directly affect OF_center_.

It should be noted that for small fields, the field output correction factor, $k_{Q_{clin},Q_{msr}}^{f_{clin},f_{msr}}$, should be applied to the raw charge for each detector to result in a true small field OF (ratio of doses) per TRS‐483.^24^ There are small field correction factors published for the microDiamond and Semiflex 3D detectors in a 1.5 T field,^25^, ^26^ but none published for the EDGE detector. The correction factors were not calculated for the EDGE for this work due to the perturbances of the EDGE to the dose distribution at small field sizes, rendering OF measurement with the EDGE less useful.

The Trufix system is designed to place the center of a given detector model at the radiation isocenter. The Trufix assumes that the active detector element is perfectly aligned with respect to the outer dimensions of the detector according to manufacturing diagrams. However, this work demonstrated that active volume positioning can vary (∼0.6 mm) from detector to detector. It should be noted that the manufacturing dates among the investigated detectors spanned around 10 years. In non‐MR environments, this effect is removed by centering the detector based on measured profiles. Only a single detector was investigated for the microDiamond therefore no conclusions can be made about active volume centering consistency for that detector.

## 5 Conclusion

In this work, the performance of the EDGE detector was compared to other small field detectors for characterizing relative dosimetry in small fields with magnetic fields. The EDGE detector was shown to under‐represent the lateral shift and shape change of crossline profiles due to the magnetic field, which is consistent with findings on other diode type detectors in the literature. Comparison with radiochromic film and MC indicated the microDiamond to be the most suitable scanning detector of those investigated. The Semiflex 3D was shown to have volume‐averaging and lateral shifts that misrepresent the profiles for small fields.

## CONFLICT OF INTEREST STATEMENT

Daniel Hyer discloses a consulting relationship with Elekta and research funding from Elekta unrelated to this work. Joel St‐Aubin discloses research funding from Elekta unrelated to this work. The remaining authors have no conflicts of interest to disclose. Sun Nuclear provided detectors for use in this work and funding for the construction of the EDGE Trufix adapter.
